# Lipid Nanoparticles Delivering Constitutively Active STING mRNA to Stimulate Antitumor Immunity

**DOI:** 10.3390/ijms232314504

**Published:** 2022-11-22

**Authors:** Wei Liu, Mohamad-Gabriel Alameh, June F. Yang, Jonathan R. Xu, Paulo J. C. Lin, Ying K. Tam, Drew Weissman, Jianxin You

**Affiliations:** 1 Department of Microbiology, Perelman School of Medicine, University of Pennsylvania, Philadelphia, PA 19104, USA; 2 Division of Infectious Diseases, Perelman School of Medicine, University of Pennsylvania, Philadelphia, PA 19104, USA; 3 Acuitas Therapeutics, Vancouver, BC V6T 1Z3, Canada

**Keywords:** lipid nanoparticles, constitutively active STING, mRNA, therapeutic approach, cancer

## Abstract

Treating immunosuppressive tumors represents a major challenge in cancer therapies. Activation of STING signaling has shown remarkable potential to invigorate the immunologically “cold” tumor microenvironment (TME). However, we have shown that STING is silenced in many human cancers, including pancreatic ductal adenocarcinoma (PDAC) and Merkel cell carcinoma (MCC). In this study, we demonstrated that mRNA-lipid nanoparticle (LNP) technology could be used to efficiently deliver naturally occurring constitutively active STING mutant STING^R284S^ into these cancer cells to reactivate STING antitumor immunity and trigger robust killing of tumor cells. STING agonists are being actively pursued as cancer immunotherapies. However, traditional STING agonists can induce T cell cytotoxicity, counteracting the desired antitumor immune response. In addition, the antitumor efficacy of traditional STING agonists obligatorily depends on STING expression and does not work in STING-silenced cancers. Importantly, we found that STING^R284S^ mRNA-LNP does not introduce T cell cytotoxicity. Our studies demonstrated that mRNA-LNP delivery of STING^R284S^ can reactivate the antitumor response without introducing antiproliferative effects in lymphocytic immune cells, overcoming the toxicity and limitations of conventional STING agonists. Our work therefore identifies a novel therapeutic tool for reactivating antitumor immunity in an array of STING-silenced immunologically “cold” tumors that are refractory to current therapies.

## 1. Introduction

Tumor immune suppression represents a major obstacle in achieving effective cancer immunotherapy against many human malignancies, including pancreatic cancer. Pancreatic cancer causes the death of around 430,000 patients per year and persists as one of the deadliest malignancies in the world [1,2,3]. Few effective treatments are available for patients with advanced pancreatic cancer [4]. Nearly 98% of pancreatic cancer patients are also resistant to PD-1/PD-L1 immune checkpoint blockade therapies [5,6,7]. Thus, there is a significant unmet need for developing more effective therapies targeting this highly lethal cancer.

Pancreatic cancer often establishes a highly immunosuppressive TME, which hinders retaliation by the host immune system and resists immunotherapies [2,8]. Therefore, cases of this cancer are traditionally classified as non-immunogenic “cold” tumors [2,8]. Typically, tumor-infiltrating CD8+ cytotoxic T cells are strongly associated with patient survival. However, the majority of pancreatic cancers lack successful infiltration of effective CD8+ T cells in the TME [9,10]. Poor intratumoral T cell infiltration and activation present a major hurdle for developing effective immunotherapies.

In our previous studies, we discovered that STING repression is a key factor underpinning the immunologically “cold” TME of MCC [11], another highly aggressive cancer with over 30% of patients showing metastatic disease at first presentation [12,13]. STING is a key regulator of innate immune signaling and antitumor responses [14,15,16,17]. The canonical role of the STING signaling pathway is to sense cytoplasmic double-stranded DNA (dsDNA), including host cytoplasmatic chromatin, mitochondrial DNA and foreign DNA such as viral DNA. These DNA molecules are recognized by cyclic GMP-AMP synthase (cGAS), which in turn synthesizes 2′3′-cGAMP that can bind to and activate STING. After stimulation by pathogen- or damage-associated molecular patterns (PAMPs or DAMPs), STING activates the transcription of type I and III interferons (IFNs) and other pro-inflammatory cytokines to initiate the innate immune response [18,19]. Cancer cells often maintain abundant damaged DNA, which can also stimulate STING-dependent induction of IFNs and other anti-tumor cytokines/chemokines, including CXCL10 and CCL5 [14,17,18]. Among the molecules activated by STING signaling, IFNs can stimulate the generation of anti-tumor T cells, T-cell infiltration and the direct killing of cancer cells [20,21,22], whereas CXCL10 and CCL5 are important for recruiting tumor-reactive effector T cells [14,15,16,23,24,25]. Therefore, activation of the STING signaling pathway in tumors can switch the TME from an immune suppressed “cold” condition to an immune activated “hot” environment [14,15,16,17,24,25,26,27,28,29,30,31].

We recently discovered that STING is silenced in MCC and that reactivating STING stimulates antitumor inflammatory cytokine/chemokine production, cytotoxic T cell infiltration and activation and eradication of MCC cells [11]. Our studies provide proof-of-principle support for the hypothesis that targeted reactivation of STING can bolster antitumor cytotoxicity and invigorate the immune-dampened TME in STING-silenced and immunologically “cold” tumors. We also found that STING is silenced or downregulated in a number of other cancers, such as PDAC [11]. In this study, we set out to develop new strategies to reactivate STING signaling in order to bolster antitumor immunity and enforce tumor immunogenicity in PDAC.

Several STING agonists have been developed to stimulate anti-tumor immune responses [16,27,31,32,33]. However, clinical trials of these STING agonists did not show beneficial results [34,35]. To overcome the limitations of traditional STING agonists, which do not work in STING-silenced cancers [31,36,37], we explored the idea of introducing naturally occurring constitutively active gain-of-function STING mutants [38,39] to reactivate antitumor immunity in STING-silenced, immunologically “cold” PDAC. STING gain-of-function mutations have emerged in multiple systemic autoinflammatory diseases, including STING-associated vasculopathy with onset in infancy, systemic lupus erythematosus-like syndromes and familial chilblain lupus diseases [38,39,40,41,42,43,44,45,46,47,48]. These mutations support constitutively hyperactive STING activity, which induces an excessive IFN response that attracts proinflammatory cells to cause autoimmune disease symptoms [38,41,43,44,45,46,47,48]. We therefore tested whether these gain-of-function STING (“hot” STING) mutants could be leveraged to “heat up” STING-deficient immunologically “cold” cancers.

We first discovered that expression of the “hot” STING^R284S^ mutant in PDAC cells robustly activates the STING signaling pathway. Using lipid nanoparticles (LNP) to deliver STING^R284S^ mRNA into cells, we observed that STING^R284S^ expression could vigorously reactivate anti-tumor cytokine production and induce cancer cell death in STING-silenced PDAC and MCC cells. Moreover, STING^R284S^ mRNA-LNP does not introduce T cell cytotoxicity, which could normally be induced by traditional STING agonists. Our results suggest that STING^R284S^ mRNA-LNP can overcome the toxicity and limitations of conventional STING agonists and therefore could be exploited as a new therapeutic approach for treating an array of STING-deficient cancers that are refractory to current therapies.

## 2. Results

### 2.1. STING Is Downregulated in Some PDAC Lesions

**Figure 1:**
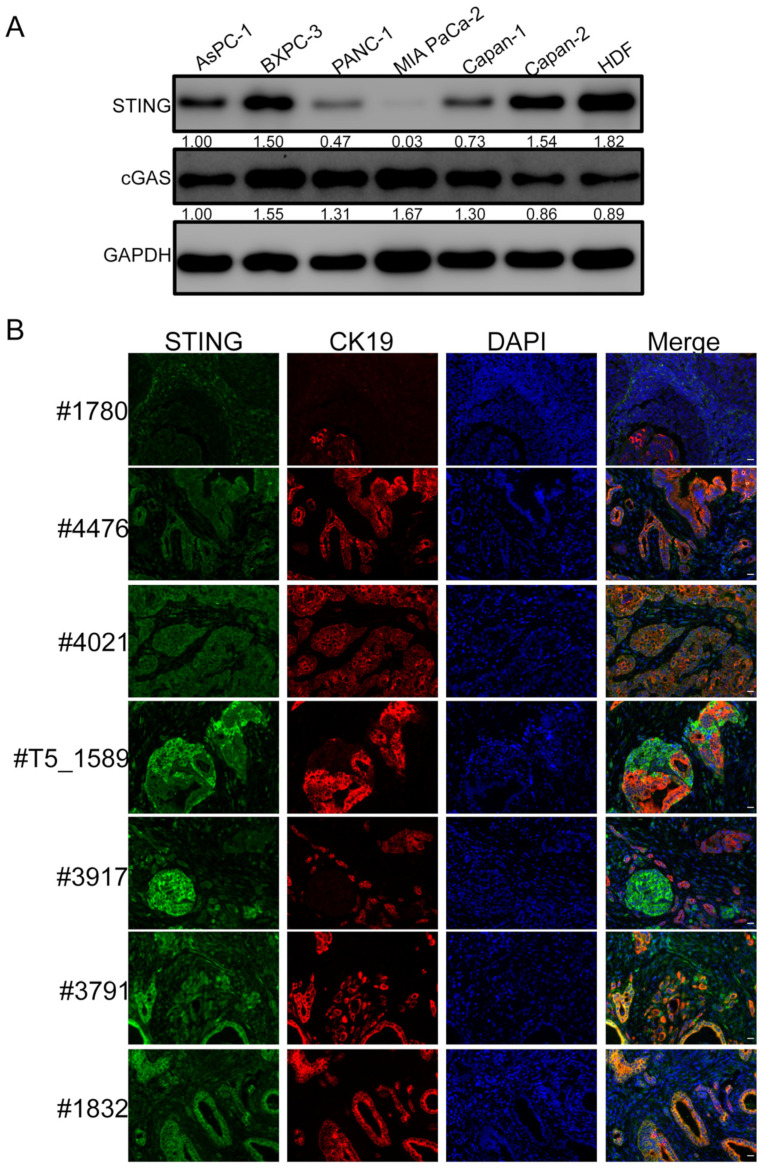
STING is downregulated in PDAC. (**A**) Whole-cell lysates of PDAC and primary HDF cells were immunoblotted using the indicated antibodies. GAPDH was used as a loading control. The protein band intensities were quantified using ImageJ software and denoted below each blot. (**B**) PDAC lesions were stained for STING (Green) and CK19 (Red), and they were counterstained with DAPI. Shown are the staining results of pancreatic lesions derived from seven different patients. Scale bar: 20 µm.

We recently discovered that STING expression is absent in MCC and several other cancer cells [11]. Following up on that study, we analyzed the STING protein levels in several PDAC cell lines and patient lesions (Figure 1). We found that STING protein is scarce in AsPC-1, PANC-1 and Capan-1 cells and virtually undetectable in MIA PaCa-2, as compared with primary human dermal fibroblasts (HDFs). In contrast, the levels of cyclic GMP-AMP synthase (cGAS), the upstream activator of STING, are clearly detected in all the tested cell lines (Figure 1A). To confirm these observations, we co-stained STING protein and the PDAC marker CK19 to examine the STING protein levels in PDAC tumor lesions. STING was nearly untraceable in three out of the seven lesions, including those from patients #1780, #4476 and #4021. An interesting observation was made for the lesions isolated from patients #T5_1589 and #3917: while STING was detected in CK19^−^ cells, it was found to be specifically silenced in CK19^+^ cells (Figure 1B). The rest of the PDAC lesions (from patient #3791 and patient #1832) show normal STING protein levels (Figure 1B). These results demonstrate that STING expression could be silenced or repressed in certain PDACs and that there appears to be a pattern of tumor cell-specific repression in some PDAC lesions. Our finding suggests that STING downregulation may contribute to the immunologically “cold” TME in some PDACs.

### 2.2. Identification of a Highly Active STING Gain-of-Function Mutant

**Figure 2:**
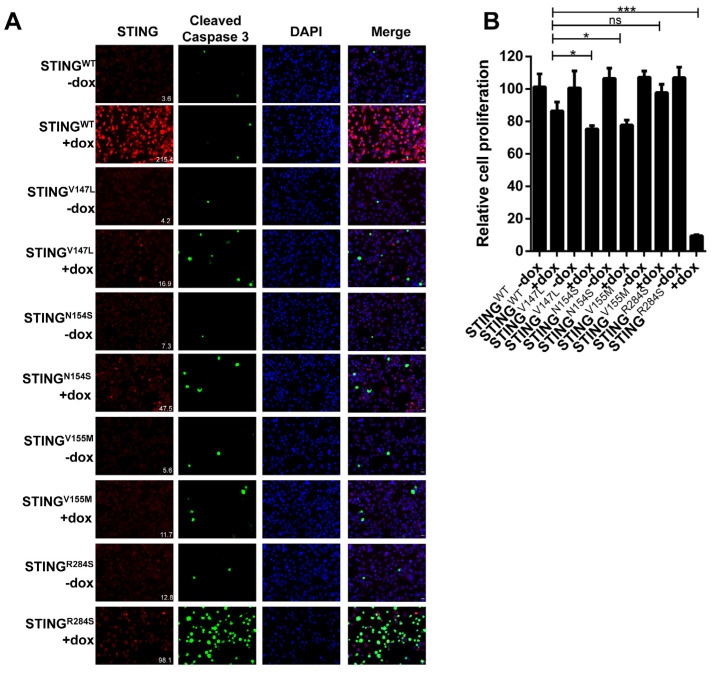
Identification of highly effective STING gain-of-function mutants. (**A**) MIA PaCa-2 cells stably expressing STING^WT^, STING^V147L^, STING^N154S^, STING^V155M^ or STING^R284S^ were treated with or without 5 µg/mL dox for 48 h. The cells were stained for STING (Red) and Cleaved Caspase-3 (Green). The mean fluorescence intensity of the STING images was quantified using ImageJ software and denoted at the bottom right corner. Scale bar: 20 µm. (**B**) MIA PaCa-2 cells stably expressing STING^WT^, STING^V147L^, STING^N154S^, STING^V155M^, or STING^R284S^ were treated with or without 5 µg/mL dox. At 96 h post-treatment, cell viability was measured by the CellTiter-Glo 3D cell viability assay (ns: not significant, * *p* < 0.05, *** *p* < 0.001).

We then set out to establish a new approach for reactivating the STING signaling pathway in STING-silenced cancers using STING gain-of-function genetic mutants. Several single amino acid STING gain-of-function mutants have been identified in autoinflammatory diseases. Among these, STING^V147L^, STING^N154S^, STING^V155M^ and STING^R284S^ mutants have demonstrated high activity in stimulating downstream innate immune signaling [38,39,48]. We therefore tested whether these gain-of-function mutants could be used to reignite the antitumor activities of the STING signaling pathway in cancer cells. To screen the capability of these STING gain-of-function mutants in blocking tumor proliferation, we constructed MIA PaCa-2 cells stably expressing either doxycycline (dox)-inducible wild-type (WT) STING or one of the STING mutants. Expression of the STING^R284S^ mutant in MIA PaCa-2 cells significantly increased the expression of the early cell death marker cleaved caspase-3 and also inhibited cell proliferation (Figure 2A,B). In contrast, expression of STING^WT^ and the other STING gain-of-function mutants did not induce such an effect (Figure 2). Notably, all the STING gain-of-function mutants showed a lower signal than WT STING (Figure 2A). This is consistent with previous studies showing that activated STING proteins are quickly degraded [49,50,51]. Based on the result of this experiment, we selected the STING^R284S^ mutant for our further studies.

### 2.3. Ectopic Expression of Dox-Inducible STING^R284S^ Induces Key Anti-Tumor Cytokine Production and Cell Death in PDAC Cells

**Figure 3:**
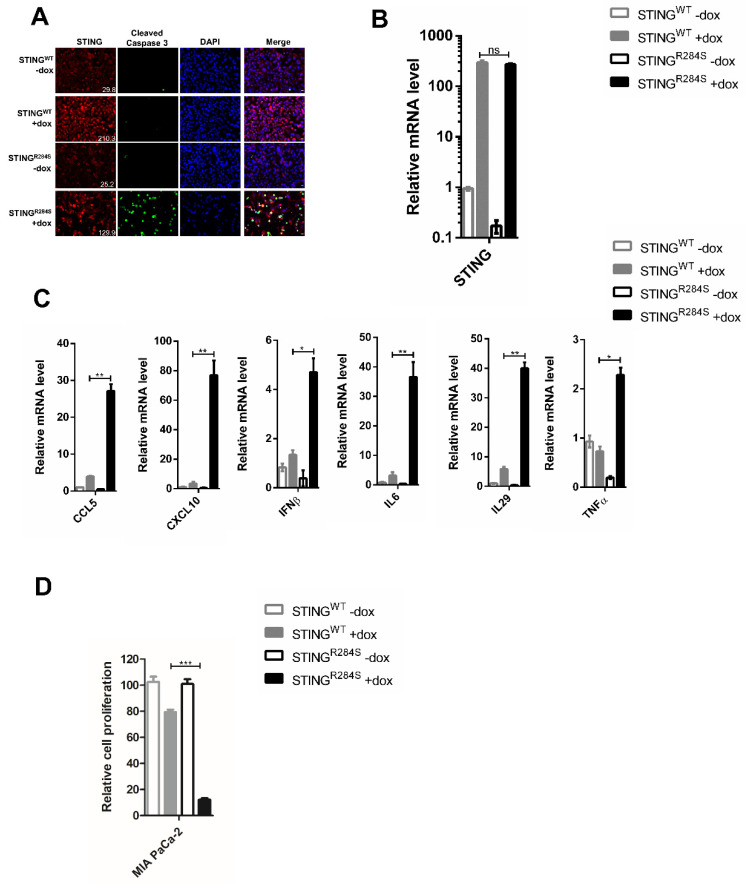
Ectopic expression of dox-inducible STING^R284S^ induces key antitumor cytokine production and cell death in PDAC cells. (**A**–**C**) MIA PaCa-2 cells stably expressing STING^WT^ or STING^R284S^ were treated with or without 5 µg/mL dox for 48 h. (**A**) The cells were stained for STING (Red) and Cleaved Caspase-3 (Green). The mean fluorescence intensity of the STING images was quantified using ImageJ software and denoted at the bottom right corner. Scale bar: 20 µm. (**B**) STING^WT^ and STING^R284S^ expression were confirmed by RT-qPCR. (**C**) The mRNA levels of the indicated genes were measured by RT-qPCR and normalized to GAPDH mRNA levels. The values for untreated STING^WT^ cells were set to 1. (**D**) MIA PaCa-2 cells stably expressing STING^WT^ or STING^R284S^ were treated with or without 5 µg/mL dox. At 96 h post-treatment, cell viability was measured by the CellTiter-GLO 3D cell viability assay. Error bars represent the SEM of three independent experiments. (ns: not significant, * *p* < 0.05, ** *p* < 0.01, *** *p* < 0.001).

Our previous study showed that reactivation of the STING signaling pathway not only induces cell death but also generates robust expression of anti-tumor cytokines, such as IFNs, CXCL10, CCL5 and IL6 [11]. To examine whether the STING^R284S^ mutant has the same downstream function, we constructed PDAC cell lines MIA PaCa-2 and BxPC-3 stably expressing dox-inducible STING^WT^ or STING^R284S^. STING expression was efficiently induced in both stable cell lines by dox treatment (Figure 3A,B and Figure S1A,B). Compared to STING^WT^, dox-induced STING^R284S^ stimulated the expression of STING’s downstream anti-tumor cytokines, such as CCL5, CXCL10, IL29, IL6, IFNβ and TNFα (Figure 3C and Figure S1C). Moreover, compared to uninduced cells and cells expressing STING^WT^, the expression of STING^R284S^ increased the level of cleaved caspase-3 and drastically inhibited the proliferation of these cancer cells (Figure 3A,D and Figure S1A,D). These results demonstrate that the STING^R284S^ mutant can provoke key anti-tumor cytokine production and cause widespread PDAC cancer cell death. In the in vivo setting, tumor cells killed by STING^R284S^ expression could release significant quantities of tumor antigens as well as DNA to activate T cells and amplify both innate and adaptive antitumor responses [52]. Our findings therefore indicate that introducing STING^R284S^ into tumor cells may be a viable therapeutic strategy for treating STING-deficient cancers.

### 2.4. A Novel Approach to Reactivate the STING Signaling Pathway

**Figure 4:**
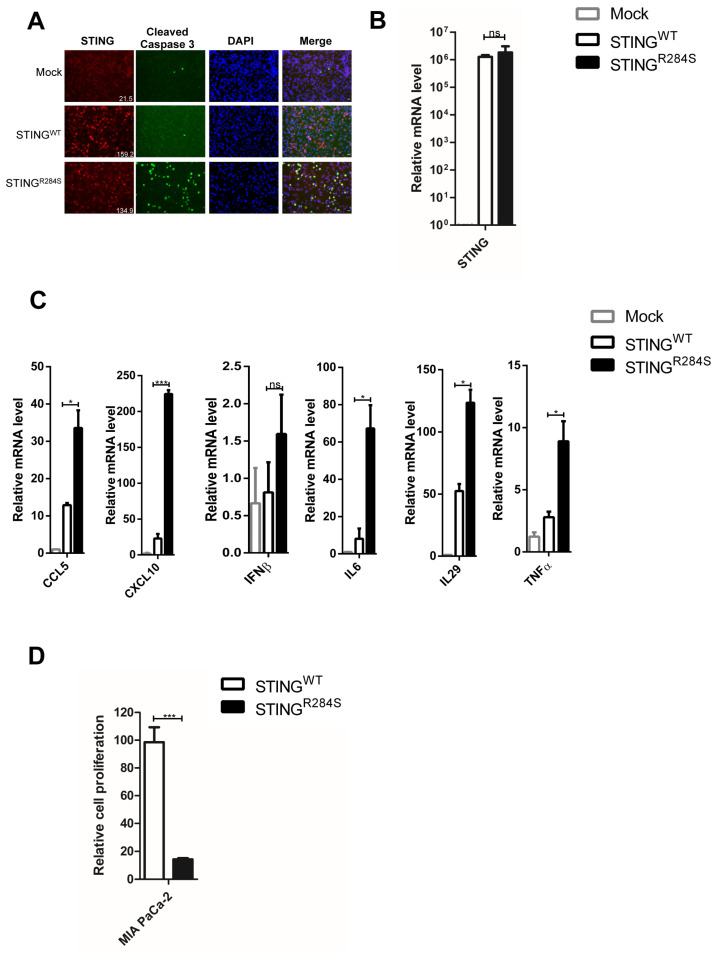
Transfection of STING^R284S^ mRNA activates vital antitumor cytokine production and triggers PDAC cell death. (**A**–**C**) 10e^4^ MIA PaCa-2 cells were transfected with 0.5 µg STING^WT^ or STING^R284S^ mRNA. Untreated MIA PaCa-2 cells were used as a negative control (mock). At 15 h post-transfection, cells were stained for STING (Red) and Cleaved Caspase-3 (Green) Scale bar: 20 µm. (**A**), STING^WT^ and STING^R284S^ expression were confirmed by RT-qPCR (**B**), and the mRNA levels of the indicated genes were measured by RT-qPCR and normalized to the GAPDH mRNA level (**C**). In (**A**), the mean fluorescence intensity of the STING images was quantified using ImageJ software and denoted at the bottom right corner. The values for untreated cells (Mock) were set to 1. (**D**) 0.5 × 10e^4^ MIA PaCa-2 cells were transfected with 1 µg STING^WT^ or STING^R284S^ mRNA. At 15 h post-transfection, cell viability was measured by the CellTiter-GLO 3D cell viability assay. Error bars represent the SEM of three independent experiments. (ns: not significant, * *p* < 0.05, *** *p* < 0.001).

We faced a challenge when trying to deliver the STING^R284S^ mutant into tumor cells as an anticancer therapeutic agent. Viral vectors cannot be used to carry the “hot” STING^R284^ mutant because activation of the STING signaling pathway blocks packaging of many viral-derived vectors [50,53,54]. On the other hand, mRNA-LNP has emerged as a powerful tool for delivering gene expression in cancer cells [55] and also as a strong T Helper 1 (Th1)-biased adjuvant [56]. Importantly, nucleoside-modified mRNA-LNP can quickly produce abundant protein in target cells while avoiding the host’s innate immune response [57,58,59,60,61]. Moreover, LNP can be used to package the “hot” STING^R284S^ mutant mRNA in vitro without activation of the host STING signaling pathway. As the first step to test this strategy, we generated mRNAs encoding STING^R284S^ and STING^WT^ and transfected them into PDAC cells. Compared to mock-transfected cells, robust STING expression was detected in STING^WT^ and STING^R284S^ mRNA-transfected PDAC cells (Figure 4A,B and Figure S2A,B). However, only STING^R284S^ mRNA, not STING^WT^ mRNA, stimulated the production of anti-tumor cytokines, such as CCL5, CXCL10, IL29, IL6, IFNβ and TNFα (Figure 4C and Figure S2C). In addition, unlike STING^WT^ mRNA, transfection of STING^R284S^ mRNA significantly elevated the level of cleaved caspase-3 and reduced the cancer cell proliferation rate appreciably (Figure 4A,D and Figure S2A,D). These results show that transfection with STING^R284S^ mRNA can specifically stimulate the STING signaling pathway to produce essential anti-tumor cytokines and kill cancer cells.

### 2.5. STING^R284S^ Expression Delivered by mRNA-LNP Activates Vital Antitumor Cytokines and Induces PDAC Cell Death

**Figure 5:**
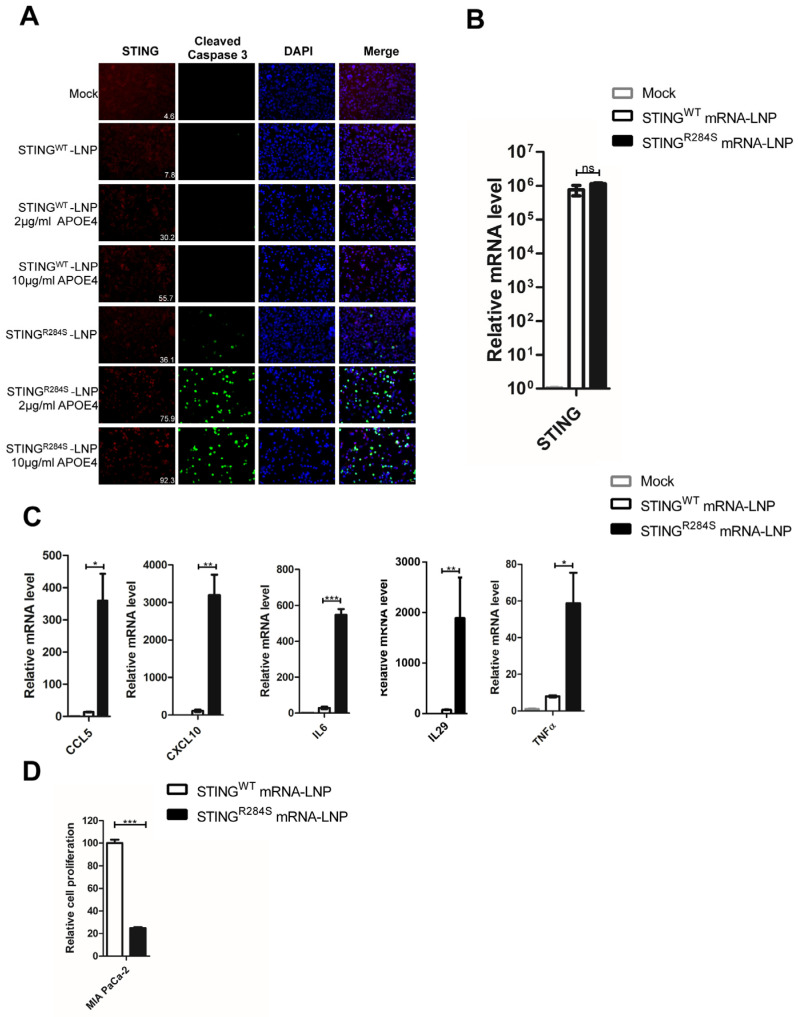
STING^R284S^ delivered by mRNA-LNP activates essential antitumor cytokine production and kills PDAC cells. (**A**) 2 × 10e^4^ MIA PaCa-2 cells were treated with 1 µg STING^WT^ or STING^R284S^ mRNA-LNP, which were pre-mixed with the indicated concentration of the recombinant human APOE4 protein. Untreated MIA PaCa-2 cells were used as a negative control (mock). At 16 h post-treatment, cells were stained for STING (Red) and Cleaved Caspase-3 (Green). The mean fluorescence intensity of the STING images was quantified using ImageJ software and denoted at the bottom right corner. Scale bar: 20 µm. (**B**,**C**) 10e^4^ MIA PaCa-2 cells were treated as in (**A**) using 10 µg/mL human APOE4 protein. At 16 h post-treatment, STING^WT^ and STING^R284S^ expression were confirmed by RT-qPCR (**B**), and the mRNA levels of the indicated genes were measured by RT-qPCR and normalized to the GAPDH mRNA level (**C**). The values for untreated cells (Mock) were set to 1. (**D**) 0.5 × 10e^4^ MIA PaCa-2 cells were treated as in (**B**). At 16 h post-treatment, cell viability was measured by the CellTiter-GLO 3D cell viability assay. Error bars represent the SEM of three independent experiments. (ns: not significant, * *p* < 0.05, ** *p* < 0.01, *** *p* < 0.001).

To further develop a therapeutic approach, we tested whether LNP could be used to deliver the STING^R284S^ mRNA into cancer cells. The LNP we exploited in this study has been shown to efficiently deliver genes in vivo [62]. However, we did not observe significant expression of STING^WT^ and STING^R284S^ in MIA PaCa-2 and BxPC-3 cells treated with the respective mRNA-LNP (Figure 5A and Figure S3A, rows 2, 5). We reasoned that this could be due to a lack of human apolipoprotein E (APOE) in our in vitro cultures. In the in vivo setting, APOE plays an important role in the cellular uptake of physiological lipoproteins through binding to low-density lipoprotein (LDL) receptors [63,64]. When mixed with mRNA-LNP before transduction, human APOE4 has been shown to radically increase mRNA-LNP transduction efficiency in vitro [63,64]. Previous studies have shown that the mRNA expression of the APOE4 receptor, lipoprotein receptor-related protein 1 (LRP1), was significantly up-regulated in PDAC tumors compared to normal pancreatic tissues [65], supporting the idea of using APOE4 to enhance the mRNA-LNP delivery efficiency. We therefore tested whether mixing STING^WT^ or STING^R284S^ mRNA-LNP with APOE4 could facilitate the delivery of mRNA into PDAC cells. We found that APOE4 robustly stimulates the delivery of mRNA-LNP into PDAC cells in a dose-dependent manner (Figure 5A and Figure S3A). Compared to untreated cells, higher levels of STING^WT^ and STING^R284S^ mRNA were detected by RT-PCR in cells treated with a combination of mRNA-LNP and APOE4 (Figure 5B and Figure S3B). The combined treatment of STING^R284S^ mRNA-LNP and APOE4 also significantly augmented the expression of key anti-tumor cytokines, such as CCL5, CXCL10, IL29, IL6 and TNFα, as compared with treatment using STING^WT^ mRNA (Figure 5C and Figure S3C). Moreover, LNP-delivered STING^R284S^ mRNA not only induced the production of cleaved caspase-3 in PDAC cells but also significantly inhibited the proliferation of these cells (Figure 5A,D and Figure S3A,D). These results demonstrate that human APOE4 can efficiently promote the delivery of mRNA-LNP into target cells, allowing the robust expression of “hot” STING^R284S^ to induce essential anti-tumor cytokines and eradicate cancer cells.

Previous studies have shown that overstimulation of STING in mice can cause antiproliferative effects and cell death in T cells and myeloid cells [14,66,67,68,69,70]. We therefore examined how the combined treatment of STING^R284S^ mRNA-LNP and APOE4 affects the viability of these immune cells. Importantly, we found that the dual treatment did not repress the proliferation of CD8+ T cells (Figure S3E). On the other hand, mRNA-LNP-delivered STING^R284S^ expression could significantly inhibit the proliferation of macrophages (Figure S4) (see discussion).

### 2.6. STING^R284S^ mRNA-LNP Also Triggers Vital Anti-Tumor Cytokine Production and Cell Death in MCC Cells

We recently found that STING is also silenced in some MCC tumors [11]. 80% of MCCs have integrated Merkel cell polyomavirus (MCPyV) genomes [71]. Our previous studies showed that STING is specifically repressed in MCPyV^+^ MCC cell lines [11]. By analyzing published RNA-seq data [72], we discovered that while STING is amply expressed in the MCPyV^-^ MCC cell line UISO, STING RNA levels are nearly undetectable in all six classic MCPyV^+^ MCC cell lines: MKL-1, MKL-2, MS-1, WaGa, PeTa and BroLi (Figure S5A). The RNA-seq data also indicated that, when compared with other MCPyV^+^ MCC cell lines, STING RNA expression is slightly higher in PeTa cells (Figure S5A) [72]. However, Western-blot analysis reveals that, similar to MKL-1 cells, STING protein expression in PeTa cells is completely imperceptible (Figure S5B). This study therefore confirmed that STING expression is suppressed in all of the classic MCPyV^+^ MCC cell lines we have examined.

**Figure 6:**
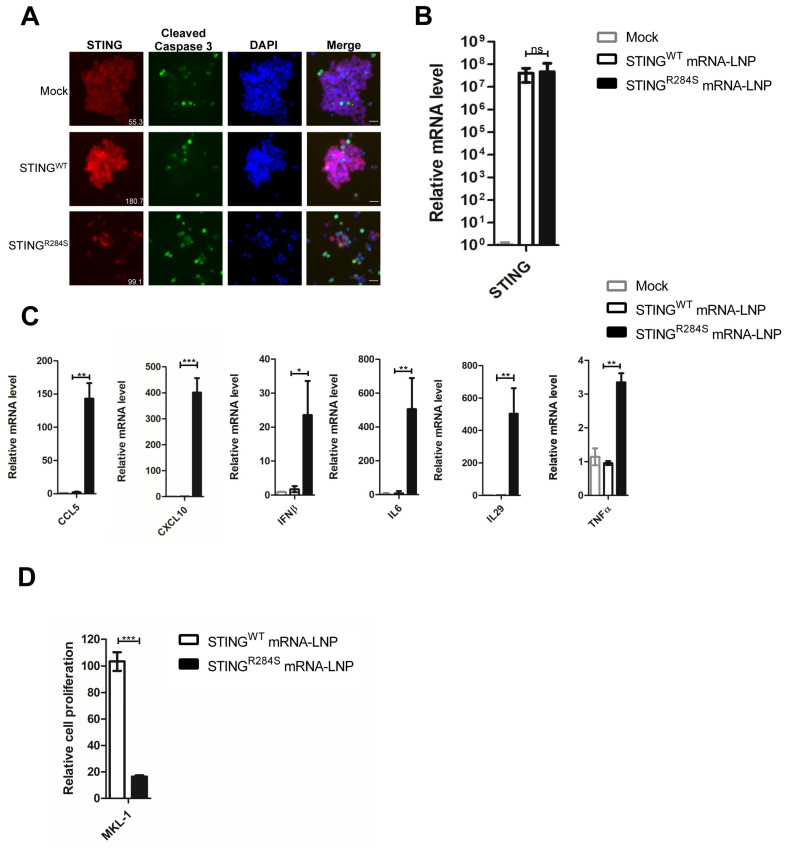
STING^R284S^ mRNA-LNP can trigger vital antitumor cytokine production and cell death in MCC cells. (**A**) 10e^4^ MKL-1 cells were treated with 1 µg STING^WT^ or STING^R284S^ mRNA-LNP, which were pre-mixed with 10 µg/mL recombinant human APOE4 protein. Untreated MKL-1 cells were used as a negative control (mock). At 16h post-treatment, cells were stained for STING (Red) and Cleaved Caspase-3 (Green). The mean fluorescence intensity of the STING images was quantified using ImageJ software and denoted at the bottom right corner. Scale bar: 20 µm. (**B**,**C**) 10e^4^ MKL-1 cells were treated as in (**A**). At 16h post-treatment, STING^WT^ and STING^R284S^ expression were confirmed by RT-qPCR (**B**), and the mRNA levels of the indicated genes were measured by RT-qPCR and normalized to the GAPDH mRNA level (**C**). The values for untreated cells (Mock) were set to 1. (**D**) 0.5 × 10e^4^ MKL-1 cells were treated as in (**B**) at 0 and 24 h. At 40 h post-treatment, cell viability was measured by the CellTiter-GLO 3D cell viability assay. Error bars represent the SEM of three independent experiments. (ns: not significant, * *p* < 0.05, ** *p* < 0.01, *** *p* < 0.001).

Encouraged by the antitumor activity of STING^R284S^ mRNA-LNP observed in PDAC cells, we tested whether this approach could be applied to stimulate the same response in MCC cells. We first optimized the mRNA-LNP delivery conditions for MCC cells using firefly luciferase mRNA-LNP. Our analysis of published RNA-seq data showed that APOE-binding receptor genes are expressed at a medium to high level in MCC MKL-1 cells (Figure S6) [73], suggesting that APOE4 could also be used to enhance mRNA-LNP delivery efficiency. We further ascertained that 10 ug/mL of human APOE4 was also the ideal concentration for delivering mRNA-LNP into MCC cells (Figure S7). When compared with untreated MKL-1 and MS-1 MCC cells, robust STING expression was detected in both STING^WT^ and STING^R284S^ mRNA-LNP-treated cells (Figure 6A,B and Figure S8A,B). However, only the delivery of STING^R284S^ mRNA-LNP, and not STING^WT^ mRNA-LNP, significantly stimulated expression of the key anti-tumor cytokines CCL5, CXCL10, IL29, IL6, IFNβ and TNFα (Figure 6C and Figure S8C). Compared to STING^WT^ mRNA-LNP, treating MCC cells with STING^R284S^ mRNA-LNP also elevated the level of cleaved caspase-3 and greatly inhibited cell proliferation (Figure 6A,D and Figure S8A,D). These results demonstrated that STING^R284S^ mRNA-LNP could also induce antitumor cytokine expression and cell death in the tested MCC cell lines.

**Figure 7:**
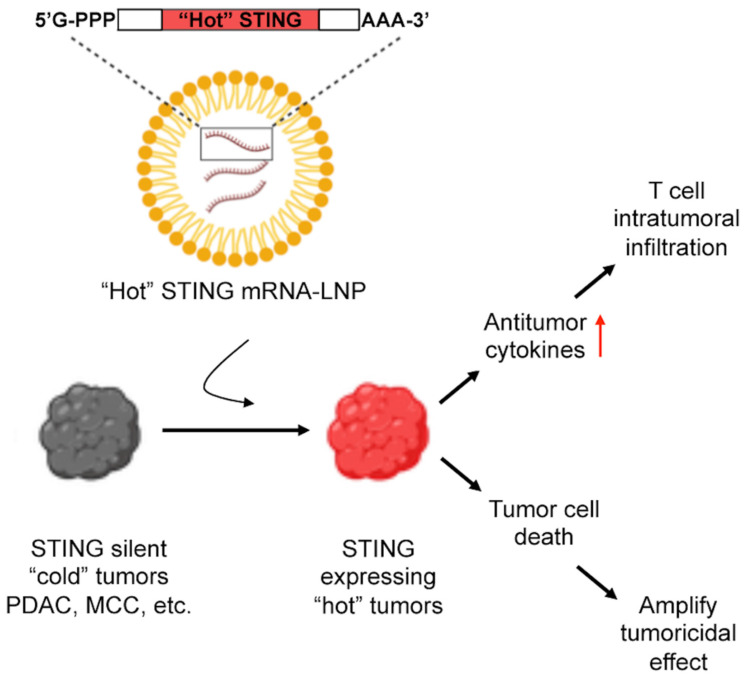
Applying “hot” STING mRNA-LNP to stimulate antitumor immunity in STING-deficient tumors. mRNA encoding constitutively active gain-of-function “hot” STING genetic mutants derived from autoinflammatory diseases can be delivered with LNP into STING silent “cold” tumors, such as PDAC and MCC, to induce STING downstream cytokines and chemokines that are crucial for promoting intratumoral infiltration of CD8^+^ T cells. Expression of “hot” STING also causes robust killing of the tumor cells, exposing tumor neoantigens to the host immune system to amplify the tumoricidal effect. This process could also stimulate adaptive antitumor immunity for rejecting distant metastases and establishing long-living immunologic memory.

In summary, we demonstrated that STING^R284S^ mRNA-LNP robustly activates the STING signaling pathway in STING-deficient cancer cells, leading to the production of key anti-tumor cytokines as well as cancer cell death (Figure 7). Therefore, STING^R284S^ mRNA-LNP could be exploited as a promising anticancer drug for treating STING-deficient cancers.

## 3. Discussion

Currently, several therapeutic approaches such as PD-1/PD-L1 and CTLA-4 inhibitors have been appraised in attempts to combat aggressive cancers such as PDACs and MCCs, but have failed to produce durable responses in PDACs [74] and led to treatment resistance in some MCCs [75]. Therefore, alternative therapeutics are still needed for treating these lethal cancers.

The complex tumor microenvironment presents a major barrier to developing broadly effective therapies. The TME of PDAC is known to be immunosuppressive. Although tumor infiltration of T lymphocytes positively correlates with overall patient survival [76], the PDAC TME has very few tumor-infiltrating CD8+ cytotoxic T cells and CD4+ T helper cells and instead exhibits an increased presence of regulatory T cells, tumor-associated macrophages and myeloid-derived suppressor cells [77,78]. We and others have reported that the STING signaling pathway is dysfunctional in several cancers [11,17,79]. Thus, we examined the expression of key components of this pathway, cGAS and STING, in PDAC cell lines. We found that all tested pancreatic cancer cell lines maintained highly expressed cGAS, but STING was significantly downregulated in many of the PDAC cell lines and tissues (Figure 1). In light of STING’s function in stimulating the antitumor response, we speculated that STING repression might contribute to the immunosuppressive TME of PDACs and that reactivating STING might represent a viable strategy for heating up the immunologically “cold” TME in PDAC.

To stimulate STING activity in PDAC cells, we first screened several “hot” STING mutants. We discovered that only the STING^R284S^ mutant, but not STING^WT^ nor the other STING gain-of-function mutants such as STING^V147L^, STING^N154S^ and STING^V155M^, could specifically inhibit the growth of STING-silenced PDAC cells (Figure 2). The result correlates appropriately with the clinical impact of these gain-of-function mutations. For example, the STING^V147L^, STING^N154S^ and STING^V155M^ mutants were identified in patients who died at an age of at least 9 years [41], but the STING^R284S^ mutant was derived from a patient who died at approximately 9 months of age [39]. Together, our finding suggests that, among all of the mutants tested, STING^R284S^ has the highest activity in stimulating the STING signaling pathway. This discovery provides the molecular basis for using the STING^R284S^ mutant to develop STING-targeted immunotherapies. The fact that the STING^R284S^ mutant can stimulate significant cancer cell death but STING^WT^ does not have such an effect is consistent with our previous finding that STING activity is the main factor driving the cancer death phenotype [11]. While our previous studies suggested that cell death may be partially caused by STING-induced cytokine-independent activities such as endoplasmic reticulum (ER) stress [11], additional studies are needed to further elucidate the underlying mechanism.

Our further studies demonstrated that STING^R284S^ mRNA-LNP could be efficiently delivered into PDAC cells to induce cytokines/chemokines crucial for promoting intratumoral infiltration of CD8+ T cells. More importantly, STING^R284S^ expression also induces robust cell death in STING-silenced cancers (Figure 5 and Figure S3). MCCs also have an immunologically “cold” TME, and STING is invariably repressed in the MCPyV+ MCC tumors we have examined. We further demonstrated that STING^R284S^ mRNA-LNP could also be utilized to activate STING downstream antitumor activity in MCC tumor cells (Figure 6 and Figure S8). In summary, by harnessing the hyperactive immuno-stimulatory activity of the STING^R284S^ mutant and the delivery capability of mRNA-LNP, we have provided evidence for using the naturally occurring STING^R284S^ mutant as a novel therapeutic tool to reactivate the antitumor response in immunologically “cold” pancreatic cancer and in other STING-silenced tumors (Figure 7).

Several observations suggest that STING^R284S^ mRNA-LNP holds great promise for developing a cancer immunotherapy. First, when compared with wild-type STING, “hot” STING mutants such as STING^R284S^ are more responsive to cGAMP [38,39,41,43]. When delivered into tumor cells by mRNA-LNP, STING^R284S^ can be further activated by the abundant damaged DNA present in these cells, spurring robust antitumoral activity. Therefore, no additional STING agonist is needed to stimulate “hot” STING mutants, increasing the feasibility of clinical application. Secondly, pancreatic cancers possess few tumor-specific new epitopes (neoantigens) [9]. STING^R284S^ mRNA-LNP-induced cell death will play a crucial role in exposing neoantigens of tumors to the host immune system. Tumor antigens released by the dead cells can be engulfed by antigen-presenting cells (APCs) and presented to T cells to generate systemic antitumor immunity and amplify the tumoricidal effect. This process could also induce adaptive antitumor immunity for rejecting distant metastases and providing long-living immunologic memory (Figure 7). Thirdly, STING^R284S^-mediated cell death can also directly reduce cancer burden, which is also clearly beneficial to cancer immunotherapy [80,81]. Finally, mRNA-LNP has an intrinsic adjuvant effect that can stimulate T follicular helper cell (Tfh) responses and promote the production of effective CD8+ T cells [56,82,83]. An additional advantage is that multiple mRNAs can be combined together or with other drugs to be encapsidated into LNP [83,84,85,86,87].

The STING^R284S^ mRNA-LNP approach can be used to restore STING expression and function in STING-deficient tumors in order to stimulate anti-tumor immune responses and directly kill the tumor cells (Figure 5C, Figure 6C, Figure 7, Figures S3C and S8C). Anti-tumor cytokines have safety concerns when systemically administered; however, gene expression driven by intratumorally injected mRNA-LNP has been detected mainly in the tumor sites but not in major vital organs [88,89]. Therefore, local delivery using STING^R284S^ mRNA formulated in LNP could overcome the specificity issue and reveal a safe approach to leverage the cytokine effects [90,91]. Additionally, overstimulation of STING in T cells could introduce cell death and cytotoxicity, which counteracts the desired antitumor immune response [14,66,67,68,69,70]. Interestingly, we found that while STING^R284S^ mRNA-LNP can effectively repress cancer cell proliferation, it does not inhibit the growth of CD8+ T cells (Figure 5D, Figure 6D, Figures S3D,E and S8D). This is consistent with previous studies confirming that T cells are not susceptible to transfection by exogenous mRNA delivered in LNP [92]. On the other hand, we found that STING^R284S^ expression delivered by mRNA LNP could significantly repress the growth of myeloid cells such as macrophages (Figure S4). Because tumor-associated macrophages play a critical role in driving the immunosuppressive TME, the STING^R284S^ mRNA LNP-induced macrophage cell death could also help to reactivate the antitumor immunity. Therefore, our findings suggest that mRNA-LNP-mediated intratumoral delivery of STING^R284S^ will allow specific activation of cancer-targeting immune responses in tumor tissues without introducing antiproliferative effects in CD8+ T cells. Because mRNA-LNP delivery is transient, it also allows for greater control of the treatment process. So far, all STING^R284S^ mRNA-LNP studies have been performed in vitro. Plans are underway to establish STING-negative tumor models in mice, which will be used to examine the efficacy of STING^R284S^ mRNA-LNP in stimulating T cell intratumoral infiltration and killing of tumor cells in vivo. Furthermore, we are also developing specific targeting strategies in order to apply STING^R284S^ mRNA-LNP for treating metastatic disease.

## 4. Materials and Methods

### 4.1. Cell Culture and Cancer Lesions

Primary foreskin dermal fibroblasts [93], human embryonic kidney 293T (HEK293T), MIA PaCa-2 and PANC-1 cells were grown in Dulbecco’s modified Eagle’s medium supplemented with 10% fetal calf serum. BxPC-3 and AsPC-1 cells were grown in RPMI 1640 medium supplemented with 10% fetal calf serum. Capan-1 and Capan-2 cells were grown in McCoy’s 5A medium supplemented with 10% fetal calf serum. MKL-1 and MS-1 cells were grown in RPMI 1640 medium supplemented with 20% fetal calf serum. Cells were incubated at 37 °C in humidified air containing 5% CO_2_. Primary CD8+ T cells from healthy donors were provided by the Human Immunology Core at the University of Pennsylvania. These cells were grown in RPMI 1640 medium supplemented with 10% heat-inactivated FBS, L-glutamine, IL-2 and penicillin–streptomycin. PDAC tissues were obtained from the Tumor Tissue and Biospecimen Bank at the University of Pennsylvania.

### 4.2. Macrophage Differentiation

Primary human monocytes from de-identified healthy donors were obtained from the Human Immunology Core at the University of Pennsylvania. The monocytes were cultured in RPMI supplemented with 10% heat-inactivated FBS, 2 mM L-glutamine, 1% Penn/Strep and 50 ng/mL recombinant human M-CSF (Gemini Bio-Products, West Sacramento, CA, USA). Macrophage differentiation was performed using a previously described protocol [94]. Specifically, monocytes were cultured in 10 mL of media in 10-cm dishes at 0.5 × 10^6^ cells per mL for 3 d; fresh media containing 50 ng/mL M-CSF was then added to the cells. The cells were cultured for an additional 3 d to complete the differentiation into macrophages.

### 4.3. Western Blot Analysis

To prepare whole cell lysates, cells were lysed in lysis buffer (10 mM HEPES, pH 7.9, 500 mM NaCl, 3 mM MgCl2, 1 mM DTT, 1 mM PMSF, 0.5% Triton X-100 supplemented with protease inhibitors). After 30 min of incubation on ice, whole cell lysates were centrifuged at 15,000× *g* for 10 min at 4 °C to remove the debris. Protein concentrations were determined using the Bradford assay. The protein samples were resolved on SDS-PAGE gels, transferred onto PVDF membranes and immunoblotted with specific primary antibodies as indicated in the figure legends. The primary antibodies used in this study include anti-STING (1:2000, 13647S, Cell Signaling Technology, Danvers, MA, USA), anti-cGAS (1:1000, 15102, Cell Signaling Technology), and anti-GAPDH (1:2000, 5174S, Cell Signaling Technology). The secondary antibody used was HRP-linked anti-rabbit IgG (1:3000, 7074S, Cell Signaling Technology). Western blots were developed using SuperSignal™ West Pico PLUS Chemiluminescent Substrate (Thermo Scientific, Waltham, MA, USA), and images were captured using a GE imaging system.

### 4.4. Cell Proliferation Assay

Cell viability was measured with CellTiter-Glo 3D (Promega, Madison, WI, USA) following the manufacturer’s instructions [95].

### 4.5. Reverse Transcription and Quantitative Real-Time PCR

Total RNA was isolated using the NucleoSpin RNA II Kit (Macherey-Nagel) in pursuance of the manufacturer’s protocol. Reverse transcription (RT) was performed using a 20 μL reaction mixture containing 350 ng of total RNA, random hexamer primers (Invitrogen, Waltham, MA, USA), dNTPs (Invitrogen) and M-MLV reverse transcriptase (Invitrogen). Quantitative real-time PCR (qPCR) was performed using a CFX96 real-time PCR detection system (Bio-Rad, Hercules, CA, USA) with IQ SYBR Green supermix (Bio-Rad). Primer sequences are the same as described previously [11]. The mRNA level of each gene was normalized to the GAPDH mRNA level.

### 4.6. Immunofluorescent Staining

Cells were fixed with 3% paraformaldehyde in PBS for 20 min. Immunofluorescent (IF) staining was performed as previously described [96]. The following primary antibodies were used: anti-CK19 (1:200, 4558, Cell Signaling Technology), anti-STING (1:500 for cell staining, 1:20 for tissue staining, 19851-1-AP, Proteintech, Rosemont, IL, USA), and anti-Cleaved Caspase-3 (Asp175) (1:500, 9661, Cell Signaling Technology). The secondary antibodies used were Alexa Fluor 594 goat anti-mouse IgG (1:500, A-11032, ThermoFisher Scientific, Waltham, MA, USA) and Alexa Fluor 488 goat anti-rabbit IgG (1:500, A-11008, ThermoFisher Scientific). All IF images were collected using an inverted fluorescence microscope (IX81; Olympus, Tokyo, Japan) connected to a high-resolution charge-coupled-device camera (FAST1394; QImaging, Surrey, BC, Canada). Images were analyzed and presented using SlideBook (version 5.0) software (Intelligent Imaging Innovations, Inc., Denver, CO, USA). The scale bars were added using ImageJ software.

### 4.7. Recombinant Plasmid Construction

The codon-optimized human STING^WT^ gene was synthesized by Genewiz. STING^R284S^ gene was generated from codon-optimized human STING^WT^ using PCR-based site-directed mutagenesis. Both of the gene fragments were subcloned into the XhoI and SpeI restriction enzyme cutting sites of the pTEV-ZIKVprM-E-A101 vector, which has been described previously [97], to replace the Zika virus sequence. The map for the new plasmid pTEV-STING-A101 is shown in Figure S9. The previously optimized 5′ UTR derived from the tobacco etch virus 5′ leader RNA and the 3′ UTR derived from Xenopus beta-globin mRNA were used to drive STING gene expression [98] (Figure S9).

### 4.8. mRNA Production

Using the linearized plasmids pTEV-STING^WT^-A101 and pTEV-STING^R284S^-A101, the STING mRNA was produced with T7 RNA polymerase. During mRNA synthesis, 1-methylpseudouridine-5′-triphosphate (TriLink, San Diego, CA, USA) was used instead of UTP to generate modified nucleoside-containing mRNA. The STING mRNA was co-transcriptionally capped using CleanCap (TriLink) and purified as described previously [56]. The STING mRNA was analyzed by agarose gel electrophoresis and stored frozen at −80 °C.

### 4.9. mRNA Transfection

Transfection of human pancreatic MIA PaCa-2 and BxPC-3 cells was performed with TransIT-mRNA (Mirus Bio, Madison, WI, USA) according to the manufacturer’s instructions. Specifically, mRNA (1 μg) was combined with TransIT-mRNA reagent (3 μL) and boost reagent (3 μL) in 100 μL of serum-free medium, and the complex was added to 10 × 10^5^ cells in 500 μL complete medium. Cells were harvested at 15–16 h after transfection.

### 4.10. LNP Encapsulation of the mRNA

Purified STING mRNAs were encapsulated in LNP using a self-assembly process in which an aqueous solution of mRNA at pH 4.0 is rapidly mixed with a solution of lipids dissolved in ethanol. The LNP used in this study was similar in composition to those described previously [97], which contain an ionizable cationic lipid (proprietary to Acuitas), phosphatidylcholine, cholesterol and PEG-lipid. The ionizable cationic lipid and LNP composition are described in the patent application WO 2017/004143. The diameter (71 to 72 nm) and polydispersity index of LNP (0.06 to 0.07) were measured by dynamic light scattering using a Zetasizer Nano ZS instrument (Malvern Instruments Ltd., Malvern, UK), and an encapsulation efficiency of 97–98% was determined using a Ribogreen assay. RNA-LNP formulations were stored at-−80 °C at an RNA concentration of ~1 μg/μL.

### 4.11. Mutagenesis Primers

The sequences for the primers used in STING mutagenesis are:

STINGV147LF: CTGTGTGAAAAAGGGAATTTCAACGTGG

STINGV147LR: TGCAGAGATCTCAGCTGGGG

STINGN154SF: AGCGTGGCCCATGGGCTGGCATGG

STINGV155MF: AACATGGCCCATGGGCTGGCATGG

STINGN154S/V155MR: GAAATTCCCTTTTTCACACACTGCAGAG

### 4.12. Statistical Analyses

Statistical analysis was performed using the unpaired t-test of GraphPad Prism software (Version 7.0) to compare the data from the control and experimental groups. A two-tailed *p* value of <0.05 was considered statistically significant.

## 5. Conclusions

STING agonists are being actively pursued as new cancer immunotherapies [31,36,37,99], but few have generated positive clinical outcomes [34,35]. As shown by our group and others, STING is silenced in many cancers [11,17,79]. Our findings could explain why traditional STING agonists will not work in these cancers, as the antitumor efficacy of these agonists obligatorily depends on STING expression to begin with [36]. When delivered into noncancerous cells, the classic STING agonists can also induce inflammatory diseases and cancers [14,70]. Our STING^R284S^ mRNA-LNP approach therefore represents a novel therapeutic strategy that could overcome the limitations and toxicity of conventional STING agonist-based therapies. It also possesses broader potential for conquering the immunosuppressive TME in other STING-silenced tumors.

## 6. Patents

Compositions and Methods for Delivering Constitutively Active Sting MRNA to Stimulate Antitumor Immunity. By Jianxin, You, et al. U.S. Provisional Patent Application 63/283,031. Filed 24 November 2021.

## Data Availability

The data obtained in this study are provided in this manuscript and in supplementary file. Additional information can be provided by corresponding authors upon request.

## Supplementary Materials

The following supporting information can be downloaded at: https://www.mdpi.com/article/10.3390/ijms232314504/s1.

## References

[1] Rawla P., Sunkara T., Gaduputi V. (2019). Epidemiology of pancreatic cancer: Global trends, etiology and risk factors. World J. Oncol..

[2] Looi C.-K., Chung F.F.-L., Leong C.-O., Wong S.-F., Rosli R., Mai C.-W. (2019). Therapeutic challenges and current immunomodulatory strategies in targeting the immunosuppressive pancreatic tumor microenvironment. J. Exp. Clin. Cancer Res..

[3] Karamitopoulou E. (2019). Tumour microenvironment of pancreatic cancer: Immune landscape is dictated by molecular and histopathological features. Br. J. Cancer.

[4] Adel N. (2019). Current treatment landscape and emerging therapies for pancreatic cancer. Am. J. Manag. Care.

[5] Feng M., Xiong G., Cao Z., Yang G., Zheng S., Song X., You L., Zheng L., Zhang T., Zhao Y. (2017). Pd-1/pd-l1 and immunotherapy for pancreatic cancer. Cancer Lett..

[6] Soares K.C., Rucki A.A., Wu A.A., Olino K., Xiao Q., Chai Y., Wamwea A., Bigelow E., Lutz E., Liu L. (2015). Pd-1/pd-l1 blockade together with vaccine therapy facilitates effector t-cell infiltration into pancreatic tumors. J. Immunother. (Hagerstown Md. 1997).

[7] Lu C., Liu K. (2017). Epigenetic regulation of pd-l1 expression and pancreatic cancer response to checkpoint immunotherapy. Transl. Cancer Res..

[8] Chang J.H., Jiang Y., Pillarisetty V.G. (2016). Role of immune cells in pancreatic cancer from bench to clinical application: An updated review. Medicine.

[9] Upadhrasta S., Zheng L. (2019). Strategies in developing immunotherapy for pancreatic cancer: Recognizing and correcting multiple immune "defects" in the tumor microenvironment. J. Clin. Med..

[10] Bazzichetto C., Conciatori F., Luchini C., Simionato F., Santoro R., Vaccaro V., Corbo V., Falcone I., Ferretti G., Cognetti F. (2020). From genetic alterations to tumor microenvironment: The ariadne’s string in pancreatic cancer. Cells.

[11] Liu W., Kim G.B., Krump N.A., Zhou Y., Riley J.L., You J. (2020). Selective reactivation of sting signaling to target merkel cell carcinoma. Proc. Natl. Acad. Sci. USA.

[12] Harms P.W., Harms K.L., Moore P.S., DeCaprio J.A., Nghiem P., Wong M.K.K., Brownell I., International Workshop on Merkel Cell Carcinoma Research Working Group (2018). The biology and treatment of merkel cell carcinoma: Current understanding and research priorities. Nat. Rev. Clin. Oncol..

[13] Becker J.C., Stang A., DeCaprio J.A., Cerroni L., Lebbe C., Veness M., Nghiem P. (2017). Merkel cell carcinoma. Nat. Rev. Dis. Prim..

[14] Barber G.N. (2015). Sting: Infection, inflammation and cancer. Nat. Rev. Immunol..

[15] Fu J., Kanne D.B., Leong M., Glickman L.H., McWhirter S.M., Lemmens E., Mechette K., Leong J.J., Lauer P., Liu W. (2015). Sting agonist formulated cancer vaccines can cure established tumors resistant to pd-1 blockade. Sci. Transl. Med..

[16] Foote J.B., Kok M., Leatherman J.M., Armstrong T.D., Marcinkowski B.C., Ojalvo L.S., Kanne D.B., Jaffee E.M., Dubensky T.W., Emens L.A. (2017). A sting agonist given with ox40 receptor and pd-l1 modulators primes immunity and reduces tumor growth in tolerized mice. Cancer Immunol. Res..

[17] Xia T., Konno H., Ahn J., Barber G.N. (2016). Deregulation of sting signaling in colorectal carcinoma constrains DNA damage responses and correlates with tumorigenesis. Cell Rep..

[18] Chen Q., Sun L., Chen Z.J. (2016). Regulation and function of the cgas-sting pathway of cytosolic DNA sensing. Nat. Immunol..

[19] Cai X., Chiu Y.H., Chen Z.J. (2014). The cgas-cgamp-sting pathway of cytosolic DNA sensing and signaling. Mol. Cell.

[20] Hubert M., Gobbini E., Couillault C., Manh T.V., Doffin A.C., Berthet J., Rodriguez C., Ollion V., Kielbassa J., Sajous C. (2020). Ifn-iii is selectively produced by cdc1 and predicts good clinical outcome in breast cancer. Sci. Immunol..

[21] Diamond M.S., Kinder M., Matsushita H., Mashayekhi M., Dunn G.P., Archambault J.M., Lee H., Arthur C.D., White J.M., Kalinke U. (2011). Type i interferon is selectively required by dendritic cells for immune rejection of tumors. J. Exp. Med..

[22] Fuertes M.B., Kacha A.K., Kline J., Woo S.R., Kranz D.M., Murphy K.M., Gajewski T.F. (2011). Host type i ifn signals are required for antitumor cd8+ t cell responses through cd8{alpha}+ dendritic cells. J. Exp. Med..

[23] Zumwalt T.J., Arnold M., Goel A., Boland C.R. (2015). Active secretion of cxcl10 and ccl5 from colorectal cancer microenvironments associates with granzymeb+ cd8+ t-cell infiltration. Oncotarget.

[24] Mowat C., Mosley S.R., Namdar A., Schiller D., Baker K. (2021). Anti-tumor immunity in mismatch repair-deficient colorectal cancers requires type i ifn-driven ccl5 and cxcl10. J. Exp. Med..

[25] Woo S.R., Fuertes M.B., Corrales L., Spranger S., Furdyna M.J., Leung M.Y., Duggan R., Wang Y., Barber G.N., Fitzgerald K.A. (2014). Sting-dependent cytosolic DNA sensing mediates innate immune recognition of immunogenic tumors. Immunity.

[26] Corrales L., Glickman L.H., McWhirter S.M., Kanne D.B., Sivick K.E., Katibah G.E., Woo S.R., Lemmens E., Banda T., Leong J.J. (2015). Direct activation of sting in the tumor microenvironment leads to potent and systemic tumor regression and immunity. Cell Rep..

[27] Lu X., Miao L., Gao W., Chen Z., McHugh K.J., Sun Y., Tochka Z., Tomasic S., Sadtler K., Hyacinthe A. (2020). Engineered plga microparticles for long-term, pulsatile release of sting agonist for cancer immunotherapy. Sci. Transl. Med..

[28] Demaria O., De Gassart A., Coso S., Gestermann N., Di Domizio J., Flatz L., Gaide O., Michielin O., Hwu P., Petrova T.V. (2015). Sting activation of tumor endothelial cells initiates spontaneous and therapeutic antitumor immunity. Proc. Natl. Acad. Sci. USA.

[29] Jing W., McAllister D., Vonderhaar E.P., Palen K., Riese M.J., Gershan J., Johnson B.D., Dwinell M.B. (2019). Sting agonist inflames the pancreatic cancer immune microenvironment and reduces tumor burden in mouse models. J. Immunother. Cancer.

[30] Wang-Bishop L., Wehbe M., Shae D., James J., Hacker B.C., Garland K., Chistov P.P., Rafat M., Balko J.M., Wilson J.T. (2020). Potent sting activation stimulates immunogenic cell death to enhance antitumor immunity in neuroblastoma. J. Immunother. Cancer.

[31] Pan B.S., Perera S.A., Piesvaux J.A., Presland J.P., Schroeder G.K., Cumming J.N., Trotter B.W., Altman M.D., Buevich A.V., Cash B. (2020). An orally available non-nucleotide sting agonist with antitumor activity. Science.

[32] Ramanjulu J.M., Pesiridis G.S., Yang J., Concha N., Singhaus R., Zhang S.Y., Tran J.L., Moore P., Lehmann S., Eberl H.C. (2018). Design of amidobenzimidazole sting receptor agonists with systemic activity. Nature.

[33] Sivick K.E., Desbien A.L., Glickman L.H., Reiner G.L., Corrales L., Surh N.H., Hudson T.E., Vu U.T., Francica B.J., Banda T. (2018). Magnitude of therapeutic sting activation determines cd8(+) t cell-mediated anti-tumor immunity. Cell Rep..

[34] Harrington K.J., Brody J., Ingham M., Strauss J., Cemerski S., Wang M., Tse A., Khilnani A., Marabelle A., Golan T. (2018). Preliminary results of the first-in-human (fih) study of mk-1454, an agonist of stimulator of interferon genes (sting), as monotherapy or in combination with pembrolizumab (pembro) in patients with advanced solid tumors or lymphomas. Ann. Oncol..

[35] Meric-Bernstam F., Sandhu S.K., Hamid O., Spreafico A., Kasper S., Dummer R., Shimizu T., Steeghs N., Lewis N., Talluto C.C. (2019). Phase ib study of miw815 (adu-s100) in combination with spartalizumab (pdr001) in patients (pts) with advanced/metastatic solid tumors or lymphomas. J. Clin. Oncol..

[36] Chin E.N., Yu C., Vartabedian V.F., Jia Y., Kumar M., Gamo A.M., Vernier W., Ali S.H., Kissai M., Lazar D.C. (2020). Antitumor activity of a systemic sting-activating non-nucleotide cgamp mimetic. Science.

[37] Gajewski T.F., Higgs E.F. (2020). Immunotherapy with a sting. Science.

[38] Luksch H., Stinson W.A., Platt D.J., Qian W., Kalugotla G., Miner C.A., Bennion B.G., Gerbaulet A., Rösen-Wolff A., Miner J.J. (2019). Sting-associated lung disease in mice relies on t cells but not type i interferon. J. Allergy Clin. Immunol..

[39] Konno H., Chinn I.K., Hong D., Orange J.S., Lupski J.R., Mendoza A., Pedroza L.A., Barber G.N. (2018). Pro-inflammation associated with a gain-of-function mutation (r284s) in the innate immune sensor sting. Cell Rep..

[40] Jeremiah N., Neven B., Gentili M., Callebaut I., Maschalidi S., Stolzenberg M.C., Goudin N., Frémond M.L., Nitschke P., Molina T.J. (2014). Inherited sting-activating mutation underlies a familial inflammatory syndrome with lupus-like manifestations. J. Clin. Investig..

[41] Liu Y., Jesus A.A., Marrero B., Yang D., Ramsey S.E., Sanchez G.A.M., Tenbrock K., Wittkowski H., Jones O.Y., Kuehn H.S. (2014). Activated sting in a vascular and pulmonary syndrome. N. Engl. J. Med..

[42] König N., Fiehn C., Wolf C., Schuster M., Cura Costa E., Tüngler V., Alvarez H.A., Chara O., Engel K., Goldbach-Mansky R. (2017). Familial chilblain lupus due to a gain-of-function mutation in sting. Ann. Rheum. Dis..

[43] Melki I., Rose Y., Uggenti C., Van Eyck L., Frémond M.L., Kitabayashi N., Rice G.I., Jenkinson E.M., Boulai A., Jeremiah N. (2017). Disease-associated mutations identify a novel region in human sting necessary for the control of type i interferon signaling. J. Allergy Clin. Immunol..

[44] Dobbs N., Burnaevskiy N., Chen D., Gonugunta V.K., Alto N.M., Yan N. (2015). Sting activation by translocation from the er is associated with infection and autoinflammatory disease. Cell Host Microbe.

[45] Keskitalo S., Haapaniemi E., Einarsdottir E., Rajamäki K., Heikkilä H., Ilander M., Pöyhönen M., Morgunova E., Hokynar K., Lagström S. (2019). Novel tmem173 mutation and the role of disease modifying alleles. Front. Immunol..

[46] Munoz J., Rodière M., Jeremiah N., Rieux-Laucat F., Oojageer A., Rice G.I., Rozenberg F., Crow Y.J., Bessis D. (2015). Stimulator of interferon genes-associated vasculopathy with onset in infancy: A mimic of childhood granulomatosis with polyangiitis. JAMA Dermatol..

[47] Patel S., Jin L. (2019). Tmem173 variants and potential importance to human biology and disease. Genes Immun..

[48] Tang E.D., Wang C.-Y. (2015). Single amino acid change in sting leads to constitutive active signaling. PLoS ONE.

[49] Gonugunta V.K., Sakai T., Pokatayev V., Yang K., Wu J., Dobbs N., Yan N. (2017). Trafficking-mediated sting degradation requires sorting to acidified endolysosomes and can be targeted to enhance anti-tumor response. Cell Rep..

[50] Liu W., Reyes H.M., Yang J.F., Li Y., Stewart K.M., Basil M.C., Lin S.M., Katzen J., Morrisey E.E., Weiss S.R. (2021). Activation of sting signaling pathway effectively blocks human coronavirus infection. J. Virol..

[51] Prabakaran T., Bodda C., Krapp C., Zhang B.C., Christensen M.H., Sun C., Reinert L., Cai Y., Jensen S.B., Skouboe M.K. (2018). Attenuation of cgas-sting signaling is mediated by a p62/sqstm1-dependent autophagy pathway activated by tbk1. EMBO J..

[52] Yum S., Li M., Chen Z.J. (2020). Old dogs, new trick: Classic cancer therapies activate cgas. Cell Res..

[53] Yamashiro L.H., Wilson S.C., Morrison H.M., Karalis V., Chung J.J., Chen K.J., Bateup H.S., Szpara M.L., Lee A.Y., Cox J.S. (2020). Interferon-independent sting signaling promotes resistance to hsv-1 in vivo. Nat. Commun..

[54] Gao D., Wu J., Wu Y.T., Du F., Aroh C., Yan N., Sun L., Chen Z.J. (2013). Cyclic gmp-amp synthase is an innate immune sensor of hiv and other retroviruses. Science.

[55] Mitchell M.J., Billingsley M.M., Haley R.M., Wechsler M.E., Peppas N.A., Langer R. (2021). Engineering precision nanoparticles for drug delivery. Nat. Rev. Drug Discov..

[56] Alameh M.G., Tombacz I., Bettini E., Lederer K., Sittplangkoon C., Wilmore J.R., Gaudette B.T., Soliman O.Y., Pine M., Hicks P. (2021). Lipid nanoparticles enhance the efficacy of mrna and protein subunit vaccines by inducing robust t follicular helper cell and humoral responses. Immunity.

[57] Kariko K., Buckstein M., Ni H., Weissman D. (2005). Suppression of rna recognition by toll-like receptors: The impact of nucleoside modification and the evolutionary origin of rna. Immunity.

[58] Kariko K., Weissman D. (2007). Naturally occurring nucleoside modifications suppress the immunostimulatory activity of rna: Implication for therapeutic rna development. Curr. Opin. Drug Discov. Dev..

[59] Kariko K., Muramatsu H., Welsh F.A., Ludwig J., Kato H., Akira S., Weissman D. (2008). Incorporation of pseudouridine into mrna yields superior nonimmunogenic vector with increased translational capacity and biological stability. Mol. Ther..

[60] Anderson B.R., Muramatsu H., Nallagatla S.R., Bevilacqua P.C., Sansing L.H., Weissman D., Kariko K. (2010). Incorporation of pseudouridine into mrna enhances translation by diminishing pkr activation. Nucleic Acids Res..

[61] Kariko K., Muramatsu H., Ludwig J., Weissman D. (2011). Generating the optimal mrna for therapy: Hplc purification eliminates immune activation and improves translation of nucleoside-modified, protein-encoding mrna. Nucleic Acids Res..

[62] Chaudhary N., Weissman D., Whitehead K.A. (2021). Mrna vaccines for infectious diseases: Principles, delivery and clinical translation. Nat. Rev. Drug Discov..

[63] Akinc A., Querbes W., De S., Qin J., Frank-Kamenetsky M., Jayaprakash K.N., Jayaraman M., Rajeev K.G., Cantley W.L., Dorkin J.R. (2010). Targeted delivery of rnai therapeutics with endogenous and exogenous ligand-based mechanisms. Mol. Ther..

[64] Sebastiani F., Yanez Arteta M., Lerche M., Porcar L., Lang C., Bragg R.A., Elmore C.S., Krishnamurthy V.R., Russell R.A., Darwish T. (2021). Apolipoprotein e binding drives structural and compositional rearrangement of mrna-containing lipid nanoparticles. ACS Nano.

[65] Gheysarzadeh A., Ansari A., Emami M.H., Razavi A.E., Mofid M.R. (2019). Over-expression of low-density lipoprotein receptor-related protein-1 is associated with poor prognosis and invasion in pancreatic ductal adenocarcinoma. Pancreatology.

[66] Larkin B., Ilyukha V., Sorokin M., Buzdin A., Vannier E., Poltorak A. (2017). Cutting edge: Activation of sting in t cells induces type i ifn responses and cell death. J. Immunol..

[67] Gulen M.F., Koch U., Haag S.M., Schuler F., Apetoh L., Villunger A., Radtke F., Ablasser A. (2017). Signalling strength determines proapoptotic functions of sting. Nat. Commun..

[68] Cerboni S., Jeremiah N., Gentili M., Gehrmann U., Conrad C., Stolzenberg M.C., Picard C., Neven B., Fischer A., Amigorena S. (2017). Intrinsic antiproliferative activity of the innate sensor sting in t lymphocytes. J. Exp. Med..

[69] Gaidt M.M., Ebert T.S., Chauhan D., Ramshorn K., Pinci F., Zuber S., O’Duill F., Schmid-Burgk J.L., Hoss F., Buhmann R. (2017). The DNA inflammasome in human myeloid cells is initiated by a sting-cell death program upstream of nlrp3. Cell.

[70] Ahn J., Xia T., Konno H., Konno K., Ruiz P., Barber G.N. (2014). Inflammation-driven carcinogenesis is mediated through sting. Nat. Commun..

[71] Santos-Juanes J., Fernandez-Vega I., Fuentes N., Galache C., Coto-Segura P., Vivanco B., Astudillo A., Martinez-Camblor P. (2015). Merkel cell carcinoma and merkel cell polyomavirus: A systematic review and meta-analysis. Br. J. Dermatol..

[72] Park D.E., Cheng J., McGrath J.P., Lim M.Y., Cushman C., Swanson S.K., Tillgren M.L., Paulo J.A., Gokhale P.C., Florens L. (2020). Merkel cell polyomavirus activates lsd1-mediated blockade of non-canonical baf to regulate transformation and tumorigenesis. Nat. Cell Biol..

[73] Cheng J., Park D.E., Berrios C., White E.A., Arora R., Yoon R., Branigan T., Xiao T., Westerling T., Federation A. (2017). Merkel cell polyomavirus recruits mycl to the ep400 complex to promote oncogenesis. PLoS Pathog..

[74] O’Reilly E.M., Oh D.Y., Dhani N., Renouf D.J., Lee M.A., Sun W., Fisher G., Hezel A., Chang S.C., Vlahovic G. (2019). Durvalumab with or without tremelimumab for patients with metastatic pancreatic ductal adenocarcinoma: A phase 2 randomized clinical trial. JAMA Oncol..

[75] Nghiem P., Bhatia S., Lipson E.J., Sharfman W.H., Kudchadkar R.R., Brohl A.S., Friedlander P.A., Daud A., Kluger H.M., Reddy S.A. (2021). Three-year survival, correlates and salvage therapies in patients receiving first-line pembrolizumab for advanced merkel cell carcinoma. J. Immunother. Cancer.

[76] Carstens J.L., Correa de Sampaio P., Yang D., Barua S., Wang H., Rao A., Allison J.P., LeBleu V.S., Kalluri R. (2017). Spatial computation of intratumoral t cells correlates with survival of patients with pancreatic cancer. Nat. Commun..

[77] Clark C.E., Hingorani S.R., Mick R., Combs C., Tuveson D.A., Vonderheide R.H. (2007). Dynamics of the immune reaction to pancreatic cancer from inception to invasion. Cancer Res..

[78] Karamitopoulou E. (2020). The tumor microenvironment of pancreatic cancer. Cancers.

[79] Xia T., Konno H., Barber G.N. (2016). Recurrent loss of sting signaling in melanoma correlates with susceptibility to viral oncolysis. Cancer Res..

[80] Dall’Olio F.G., Marabelle A., Caramella C., Garcia C., Aldea M., Chaput N., Robert C., Besse B. (2021). Tumour burden and efficacy of immune-checkpoint inhibitors. Nat. Rev. Clin. Oncol..

[81] Huang A.C., Postow M.A., Orlowski R.J., Mick R., Bengsch B., Manne S., Xu W., Harmon S., Giles J.R., Wenz B. (2017). T-cell invigoration to tumour burden ratio associated with anti-pd-1 response. Nature.

[82] Knudson C.J., Alves-Peixoto P., Muramatsu H., Stotesbury C., Tang L., Lin P.J.C., Tam Y.K., Weissman D., Pardi N., Sigal L.J. (2021). Lipid-nanoparticle-encapsulated mrna vaccines induce protective memory cd8 t cells against a lethal viral infection. Mol. Ther..

[83] Sahin U., Oehm P., Derhovanessian E., Jabulowsky R.A., Vormehr M., Gold M., Maurus D., Schwarck-Kokarakis D., Kuhn A.N., Omokoko T. (2020). An rna vaccine drives immunity in checkpoint-inhibitor-treated melanoma. Nature.

[84] Awasthi S., Hook L.M., Pardi N., Wang F., Myles A., Cancro M.P., Cohen G.H., Weissman D., Friedman H.M. (2019). Nucleoside-modified mrna encoding hsv-2 glycoproteins c, d, and e prevents clinical and subclinical genital herpes. Sci. Immunol..

[85] Awasthi S., Knox J.J., Desmond A., Alameh M.G., Gaudette B.T., Lubinski J.M., Naughton A., Hook L.M., Egan K.P., Tam Y.K. (2021). Trivalent nucleoside-modified mrna vaccine yields durable memory b cell protection against genital herpes in preclinical models. J. Clin. Investig..

[86] LaTourette P.C., Awasthi S., Desmond A., Pardi N., Cohen G.H., Weissman D., Friedman H.M. (2020). Protection against herpes simplex virus type 2 infection in a neonatal murine model using a trivalent nucleoside-modified mrna in lipid nanoparticle vaccine. Vaccine.

[87] Sahin U., Derhovanessian E., Miller M., Kloke B.P., Simon P., Lower M., Bukur V., Tadmor A.D., Luxemburger U., Schrors B. (2017). Personalized rna mutanome vaccines mobilize poly-specific therapeutic immunity against cancer. Nature.

[88] Munakata L., Tanimoto Y., Osa A., Meng J., Haseda Y., Naito Y., Machiyama H., Kumanogoh A., Omata D., Maruyama K. (2019). Lipid nanoparticles of type-a cpg d35 suppress tumor growth by changing tumor immune-microenvironment and activate cd8 t cells in mice. J. Control. Release.

[89] Yanagi T., Tachikawa K., Wilkie-Grantham R., Hishiki A., Nagai K., Toyonaga E., Chivukula P., Matsuzawa S. (2016). Lipid nanoparticle-mediated sirna transfer against pctaire1/pctk1/cdk16 inhibits in vivo cancer growth. Mol. Ther. Nucleic Acids.

[90] Hewitt S.L., Bai A., Bailey D., Ichikawa K., Zielinski J., Karp R., Apte A., Arnold K., Zacharek S.J., Iliou M.S. (2019). Durable anticancer immunity from intratumoral administration of il-23, il-36gamma, and ox40l mrnas. Sci. Transl. Med..

[91] Hewitt S.L., Bailey D., Zielinski J., Apte A., Musenge F., Karp R., Burke S., Garcon F., Mishra A., Gurumurthy S. (2020). Intratumoral il12 mrna therapy promotes th1 transformation of the tumor microenvironment. Clin. Cancer Res. Off. J. Am. Assoc. Cancer Res..

[92] Tombacz I., Laczko D., Shahnawaz H., Muramatsu H., Natesan A., Yadegari A., Papp T.E., Alameh M.G., Shuvaev V., Mui B.L. (2021). Highly efficient cd4+ t cell targeting and genetic recombination using engineered cd4+ cell-homing mrna-lnps. Mol. Ther..

[93] Liu W., Yang R., Payne A.S., Schowalter R.M., Spurgeon M.E., Lambert P.F., Xu X., Buck C.B., You J. (2016). Identifying the target cells and mechanisms of merkel cell polyomavirus infection. Cell Host Microbe.

[94] Casson C.N., Yu J., Reyes V.M., Taschuk F.O., Yadav A., Copenhaver A.M., Nguyen H.T., Collman R.G., Shin S. (2015). Human caspase-4 mediates noncanonical inflammasome activation against gram-negative bacterial pathogens. Proc. Natl. Acad. Sci. USA.

[95] Liu W., Krump N.A., Herlyn M., You J. (2020). Combining DNA damage induction with bcl-2 inhibition to enhance merkel cell carcinoma cytotoxicity. Biology.

[96] Liu W., Stein P., Cheng X., Yang W., Shao N.Y., Morrisey E.E., Schultz R.M., You J. (2014). Brd4 regulates nanog expression in mouse embryonic stem cells and preimplantation embryos. Cell Death Differ..

[97] Pardi N., Hogan M.J., Pelc R.S., Muramatsu H., Andersen H., DeMaso C.R., Dowd K.A., Sutherland L.L., Scearce R.M., Parks R. (2017). Zika virus protection by a single low-dose nucleoside-modified mrna vaccination. Nature.

[98] Pardi N., Muramatsu H., Weissman D., Kariko K., Rabinovich P. (2013). Synthetic Messenger RNA and Cell Metabolism Modulation. Methods in Molecular Biology (Methods and Protocols).

[99] Corrales L., Gajewski T.F. (2015). Molecular pathways: Targeting the stimulator of interferon genes (sting) in the immunotherapy of cancer. Clin. Cancer Res..

